# Identifying opportunities to strengthen school food environments in the Pacific: a case study in Samoa

**DOI:** 10.1186/s12889-021-10203-2

**Published:** 2021-01-29

**Authors:** Erica Reeve, Anne-Marie Thow, Colin Bell, Christina Soti-Ulberg, Gary Sacks

**Affiliations:** 1 Deakin University, Global Obesity Centre, Institute for Health Transformation, School of Health and Social Development, 1 Gheringhap Street, Locked Bag 20001, Geelong, Victoria 3220 Australia; 2 Menzies Centre for Health Policy, School of Public Health, University of Sydney, Level 2, Charles Perkins Centre, University of Sydney, Sydney, NSW 2006 Australia; 3 Deakin University, School of Medicine, 1 Gheringhap Street, Geelong, Victoria 3220 Australia; 4 Ministry of Health Samoa, Motootua, Ifiifi Street, Apia, Samoa

**Keywords:** Implementation lessons, School food environments, Multisectoral nutrition, Childhood obesity, Policy barriers, Policy analysis

## Abstract

**Background:**

Despite global recommendations to prioritise policies that create healthy food environments within education institutions, the implementation of effective healthy school food policies has proved challenging for many countries. This study examined the experience of Samoa subsequent to the 2012 introduction of a stronger policy to improve the healthiness of school food environments. Our aim was to identify opportunities to strengthen healthy school food policy implementation in Samoa and other comparable contexts.

**Methods:**

We used a qualitative case study approach, underpinned by policy science theory. In 2018, we conducted in-depth semi-structured interviews with 30 informants, coupled with analysis of relevant documents, to generate a detailed understanding of the relevant policy implementation processes in Samoa, and the perspectives and capacities of key implementation actors. Data collection and analysis were guided by the Health Policy Analysis Triangle, supplemented by other policy theories relevant to policy process.

**Results:**

Samoa’s school food policy operationalizes international ‘best practice’ recommendations. We found health policymakers and leaders in Samoa to be strongly committed to improving school food environments. Despite this, there continued to be challenges in ensuring compliance with the school nutrition standards. Key issues that negatively impacted the policy’s effectiveness were the lack of priority given to school food by stakeholders outside of health, the high prevalence of unhealthy food in the areas immediately surrounding schools, vendor knowledge and capacity, and the high degree of agency exercised by actors in and around the school. We noted several opportunities for policies to be effectively implemented and sustained. Respondents identified community-level leaders as potentially pivotal stakeholders, particularly where school governance arrangements draw heavily on community representation.

**Conclusions:**

Sustained and effective implementation of healthy school food policies requires continued engagement from political and community leaders, beyond initial commitment. There is potential to capitalize on political will for diet-related NCD prevention by more clearly demonstrating the institutional and operational requirements for effective and sustained implementation. Strong incentives for compliance and effective enforcement mechanisms are also likely to be crucial to success.

**Supplementary Information:**

The online version contains supplementary material available at 10.1186/s12889-021-10203-2.

## Background

Noncommunicable diseases (NCDs) are the main contributor to mortality and morbidity globally [1]. The prevention of NCDs is a key target of the Sustainable Development Goals due to the impact of NCDs on population health, livelihoods and national economies [2]. Childhood overweight and obesity are major risk factors for the development of diet-related NCDs, and increasing global prevalence is therefore of concern to policymakers [3, 4].

Schools are a setting where children’s dietary behaviours can be influenced during a formative period [5, 6]. As a result, school food policies are critical components of comprehensive action for NCD prevention [3]. The World Health Organization recommends that countries prioritise policies that create nutrition-promoting environments within education institutions [7], including by adopting standards that promote healthy food and non-alcoholic beverages (FNAB) whilst eliminating the provision, sales or marketing of unhealthy FNAB [8]. Schools that promote the consumption of healthy FNAB can contribute to food security by stimulating local food production and the local economy [9], and the public procurement of food is increasingly viewed as an opportunity to promote policy coherence [10, 11].

Schools are tightly regulated by governments, and, conceptually, should be straightforward targets for regulation regarding healthy food provision [8, 9]. However, evidence from both high-income [12–14] and low- and middle-income [15–17] countries suggests that implementing and maintaining compliance with school food standards has proved difficult. Reasons include the lack of emphasis placed on the school food environment by administrators and the broader school community, and the absence of effective enforcement mechanisms [18, 19]. The relative expense of healthy food, and a perceived lack of financial viability for implementing school nutrition standards, are frequently reported as barriers to the implementation of school food policies [20–22]. The lack of understanding of healthy food provision and school food standards, and absence of the requisite knowledge and skills to execute them is common among school-based vendors [16, 18, 19, 21, 23, 24]. Complicating factors for school food policy making in developing country contexts include that schools are situated within highly complex, and often largely unregulated, food environments [25–28], that typically include informal food vendors surrounding schools and selling predominately unhealthy food and non-alcoholic beverages [16, 25, 28–30]. The influence of informal vendors is compounded because children in lower socioeconomic settings are more likely to walk to school [31–34], thereby increasing their exposure to unhealthy alternatives.

Like many other Small Island Developing States (SIDS) [35], Samoa has experienced rapid increases in the prevalence in overweight and obesity in children [36, 37]. Faced with these challenges, the Government of Samoa has demonstrated a strong commitment to improving the diet of the Samoan population in an effort to address diet-related NCDs [38–41]. A government-led review found that the most children in Samoa did not bring food or drinks to school from home, and were fully reliant on vendors in and around the school, largely selling ‘*foods high in fat, salt and sugar and contain few nutrients (page 7)* [42]. In response to this, health leaders led a reform process resulting in the launching of a new School Nutrition Standards (‘the Standards’) in 2012. The Standards provide detailed guidance on the types of FNAB that should be restricted from being provided, promoted, sold or introduced to the school grounds [43]. The Ministry of Health is principally responsible for delivering on the Standards, under the auspice of the Health Promotion Schools (HPS) Committee comprising representatives from Health, Education, Women, and Agriculture sectors [44]. At the time the Standards where launched, policy leaders engaged parliamentarians to promote the policy, and invested substantial financial and human resources towards its adoption and implementation across all government schools [45]. However, there has been no formal investigation of how the implementation of the Standards has progressed.

The World Health Organization’s Commission for Ending Childhood Obesity calls on researchers to address knowledge gaps with regard to policy implementation [46]. Implementation is the continual process to carry out activities that are aligned to a policy’s objectives. It is shaped by diverse contexts [47–49], and led by a community of actors with varying priorities, capacities and resources [50–52]. Conflict between policy enactment and implementation often relates to a divide between government agents delegating responsibility for implementation to agents over whom they have ‘indirect’ and ‘incomplete’ control [46]. While there is plenty of evidence of barriers to operationalizing healthy food policies in the school setting, the vast majority of policy literature is from high-income settings [53]. There is a need to improve our understanding of factors influencing policy processes in resource-poor settings with reduced regulatory capacity [53, 54].

This study examined the experience of Samoa in introducing a stronger policy to improve the healthiness of school food environments. Our aim was to identify opportunities to strengthen healthy school food policy implementation in Samoa and other comparable contexts.

## Methods

### Study design

We adopted a qualitative case-study approach. This approach is well suited to the investigation of solutions to complex problems, and the exploration of relevant contextual influences [55]. Our case-study included policy analysis, which is an analytical approach that draws on theory from the political sciences literature to promote exploration of the relationship between individuals, institutions, ideas and policy processes [53]. Our data sources included semi-structured in-depth interviews with key stakeholders involved in the relevant policy development and implementation processes, and analysis of relevant policy documents. The use of multiple data sources enabled triangulation of data as part of the analysis of the case study [55]. The Consolidated Criteria for Reporting Qualitative Research (COREQ) was applied to enhance transparency [56]..

### Theoretical frameworks

We used the Health Policy Analysis Triangle (HPAT) as our primary framework to guide study design and analysis. The four constructs of the HPAT (Actors, Context, Processes and Contents) are grounded in policy theory to prompt investigation into how different aspects of the policy context affect the way a policy is developed and disseminated [53, 57, 58], the way individual and groups of actors interact with the process of policy-making and implementation [46, 51, 59–62], and the development and interpretation of policy contents. The HPAT was considered to be well aligned with our study aim regarding policy implementation in developing countries, and has been applied in similar nutrition case studies [15, 16, 63, 64]. In order to match the aim of the study, ‘Outcomes’ was added to the HPAT framework as an additional construct. As part of our analysis, we also supplemented the HPAT with concepts from other policy theories, including Sabatier’s Advocacy Coalition Framework [60], Lipsky’s Street-Level Bureaucracy [61], and Potter and Brough’s Hierarchy of Capacity Needs [51]. Key considerations from these theories were used to articulate specific codes under each construct of the HPAT (Table 1).

**Table 1:** Summary of theoretical domains adopted for data collection and analysis

Actors	Contextual factors	Processes	Content	Outcomes
• Roles of actors ▪ Institutions ▪ Government policymakers & officers ▪ Actors responsible for implementation (school leaders, vendors) • Actors exercising agency • Motivation (interests, incentives) • Capacity (resources, cognition)	• Political • Environmental • Cultural • Institutional structures and mandates • Resources	• Planning • Resources • Communication • Coordination • Monitoring • Incentives • Enforcement	• Policy clearly constructed • Specificity of policy • Evidence-based change outcome • Acknowledges risks, addresses risks • Enforceable within mandate	• Foods available in schools • Perceived healthfulness of foods in schools • Types of foods perceived as healthy • Sense of priority for school nutrition

### Interview participants

In July 2017, the lead author and in-country collaborators conducted in-depth interviews with 30 key stakeholders. Interview participants included senior policymakers (*n*=5), policy officers (*n*=8), school principals (*n*=9), school-based food vendors (n=6), a representative from a public health organization (n=1) and a politician overseeing the health portfolio (*n*=1). Informants were initially selected in consultation with the Ministry of Health (MOH) and the Ministry of Education, Sports and Culture (MESC) as being relevant to school food and nutrition promotion. Snowball sampling was used to identify additional informants viewed as relevant to the school food policymaking process across two ministries. School-level informants were selected in collaboration with MESC by nominating two urban and two rural settings on Upolu.

The researcher conducting the interviews was known to many health informants as a former public health policy advisor with a strong understanding of the policy context. Local researchers were selected from each MOH and MESC to maintain engagement in the research process and promote ownership of results. All interview participants provided informed consent.

### Interviews

Interview schedules were based on the selected theoretical domains outlined in Table 1. Questions were asked about the policy process based on the policy cycle [49] (development, implementation, monitoring, evaluation), policy context (political, cultural, socioeconomic, environmental), influences on the role and actions of different actors (capacity, knowledge, motivation) and actor agency (compliance and incentives). Interview schedules have been provided in Additional File 1.

Interviews were semi-structured, and modified based on the roles and experiences of the informant, with prompts used to provide further context or illicit different information. Interviews ranged from 10 to 50 min, depending on the richness of the information being provided and the relevance of the participant to the process. Most interviews were conducted in English apart from two, where the interview was translated into English by co-researchers. Interviews were recorded and transcribed, and field notes maintained. Verification occurred throughout the interviews, as well as by sending transcripts to all participants that provided an email address (*n*=14, all policymakers).

### Document review

Co-researchers and key informants within the MOH and MESC provided relevant documents to inform the study. These documents were analysed to provide insight into the relevant policy context, processes and policy outcomes. In total, 18 documents were analysed, including: strategy and policy frameworks (*n*=4); HPS committee meeting minutes (*n*=2); a report of the 2007 consultation review process (*n*=1); the School Nutrition Standards Guide (n=1); a workplan for a parliamentary health advocacy group (n=1); Minimum Service Standards for schools (n=1); and school health monitoring reports (*n*=6) and data collection and entry forms (*n*=2). The compliance rate underpinning policy outcomes was obtained from Ministry-generated biannual reports, based on data collected during biannual compliance visits.

### Coding and analysis

Using NVivo, we coded document and interview data against the domains from the selected theoretical frameworks (Table 1). In line with our research aim, we centred our analysis on documenting different elements of the policy processes, how they were influenced or experienced by relevant actors, and the expressed needs of these actors in supporting more successful implementation. We reviewed and summarised initial coding, then arranged codes into themes using an iterative approach based on similar or overlapping concepts and concerns. In triangulating data from the interviews and the documents, we looked for patterns of convergence or discrepancy. The final themes included three enablers of effective and sustained school food policy implementation and six key implementation challenges. Our analysis was reviewed and discussed by three members of the team.

## Results

We first present an overview of the school food policy context in Samoa, and a summary of the policy outcomes in relation to food in schools, as gleaned from our analysis. We then present an analysis of each of the enablers of effective and sustained school food policy implementation, and the six key implementation challenges. Finally, we provide a summary of the participants’ suggestions to strengthen implementation of the policy.

### Overview of the Samoan school food policy context

According to policymakers, nearly all public schools (urban and rural) are situated inside villages, and these villages take a central role in carrying-out government programs. In most areas, school operations and infrastructure are the responsibility of the surrounding village and school committee, while MESC adopts responsibility for teaching, learning and accountability in the public-school system. The arrangements for school food vendor operations vary widely between schools, though most food vendors operate on school grounds as tenants. According to policymakers, some of the vendors have a registered business permit, though it is not a requirement. At the school level, accountability to the Standards rests with the school committee.

### Evidence of policy outcomes

Interviewees from MESC reported that they believed that the introduction of the Standards in 2012 had led to vast improvements in school food environments. Some schools we visited sold a range of compliant foods, including sandwiches, eggs, popcorn, spaghetti, bags of dried cereal (e.g. rice crisps) and curry with rice. At least three principals discussed having complementary programs to promote healthy eating, for instance, by growing fruit trees or displaying nutrition promotion materials. Additionally, health officers provided examples of schools that had invited local families in the village to supply healthy soup for lunch, or fruit for morning tea, as revenue-earning opportunities.

However, Interviewees from both MOH and MESC indicated that unhealthy foods and beverages were still widely available in and around schools. This was confirmed by ER’s visual inspection at the schools, where vendors were observed selling cakes, doughnuts, packaged sweet biscuits, chips and extruded snacks, milo and other sugar-sweetened beverages, hot dogs and other processed meat franks, noodles, chop suey (noodles with soy sauce), ice-cups and white-bread sandwiches. Low compliance was confirmed by the Government’s biannual monitoring reports, which reported compliance lingering between 32 and 36% across all years between 2014 and 2018 [65, 66]. The compliance report for 2016 found that over 55% of schools still sold ice blocks and sweetened beverages, despite widespread recognition that they were a banned product for schools [66]. Conclusions from the government’s own reports acknowledge the lack of progress in improving compliance, and called on stakeholders to make greater efforts to adhere to the Standards.

### Enablers of successful implementation of school nutrition standards in Samoa

#### The standards are supported by a clear mechanism for governance and monitoring

The government delivers a coordinated monitoring program to review compliance with the Standards, through biannual visits to schools by health promotion and nutrition officers together with representatives from MESC. As a part of the monitoring program, indicators include that all food brought, sold, and procured at the school comply with the nutrition guidance, that the Standards are displayed at the school for staff, students and vendors, that canteen vendors are licensed, that there are no vendors operating around the school perimeter, and that children are not allowed to leave the premises during school hours. Monitoring visits routinely include feedback to school principals, four of whom explained that the monitoring visits were a useful mechanism to remind them of the policy.

*“So that (the monitoring visits) challenges us to make sure everything is prepared and done by the Standards given to us.” (Principal, Interview 33)*

Participants indicated that school principals are encouraged to share the results of monitoring visits with the school committee to discuss with the food vendors, staff and wider community, however, the extent to which this occurs is unknown. Biannual reports on compliance are aggregated and submitted to policy leaders in Health and Education, and the HPS Committee. Reports are also presented to schools during annual HPS Symposiums, where an award system is offered as an incentive. According to health officers, schools not complying with the Standards risk an official letter of reprimand or rebuke by political leaders.

#### The school health space is a priority of policy leaders and health officials

Our documentary analysis reflected that MOH, as the primary driver of health policy in Samoa, has continually prioritised school health throughout their policies, and allocated substantial resources to implementing HPS, including by resourcing a team of health workers to undertake year-round monitoring.

According to interviews, members of Samoa’s Parliamentary Advocacy Group for Healthy Lifestyles, a group of parliamentarians championing public health issues, had been instrumental in closing schools that were not meeting sanitation standards, and has threatened closure to those not meeting tobacco standards.

#### Education officials recognised the importance of healthy eating

All Education officials interviewed for this research indicated that they viewed nutrition as important, especially in view of the NCD crisis in Samoa, and had a strong interest in options for schools to more positively influence children’s eating behaviours. The education sector had demonstrated some commitment to improving school nutrition, for instance by participating in nutrition-promoting events and by integrating a performance indicator related to the Standards into a new set of Minimum Service Standards (2017) as an attempt to improve their enforceability.

A third of the principals we met with reported monitoring the school canteen themselves. In some cases, they had issued warnings to the canteen when non-compliance with the Standards was identified, or had reported poor compliance to the school committee. One principal had successfully encouraged the committee to integrate compliance with the Standards into the canteen vendor’s contract. The contract was subsequently terminated when the vendor was found not to comply.

*“The partnership is really important, …I’m so blessed to have a very good support from not only the parents but the school committee….I raised it to the school committee…so we have made a letter to the canteen man and cancelled his contract” (Principal, Interview 34)*

### Implementation challenges

#### The school nutrition standards are not enforceable

The Standards have no clear implications for non-compliance, and policymakers and principals consistently noted that the lack of enforceability was a significant barrier to policy implementation and effectiveness. Policy officers explained that they could only provide warnings to non-compliant schools, with no other sanctions available to them. It was evident through these interviews that the existing accountability mechanisms were exerting very little pressure on food vendors operating in and around schools. Other health policies that apply to schools and are protected by legislation (for example The Tobacco Act and Food Safety Act) were all enforceable, risking school closure for non-compliance.

*“If they sell the food in unhygienic environments, they would be warned or a closure…School Nutrition Standards, there's nothing in the law [to enforce it]…They don't take it seriously unless we regulate the food to be sold in schools.” (Health Official, Interview 15)*

Interviewees noted that many of the complementary activities intended to create nutrition-promoting environments within the school and community, for instance, the purchase and consumption of foods grown locally in the community and the engagement of children in the production and preparation of food were not being monitored or reported on.

#### Differing perceptions of responsibility for implementation

Despite substantial efforts from MOH to address the contribution of poor nutrition to NCDs, Health officials believed nutrition was a low-priority for other key actors, specifically teachers, villages and MESC. Participants from MOH indicated that the Standards would be more effective should the Education department take over responsibility for their implementation. Conversely, the Education officials that we interviewed indicated that they believed nutrition promotion to be the responsibility of Health officials.

*“As I see, because the Education ministry, they are not encouraging because they said their goal is just teaching. They are there to teach, not to do anything else about nutrition” (Senior Health Official, Interview 15)*

*“(School) nutrition should sit with MOH and they should be driving it as well. Collaborating of course with us, when they do go into schools” (Education Official, Interview 12)*

Four Health officials noted that the interests of partnering sectors had dwindled over time, and that officers representing them at committee meetings often rotated between meetings, affecting continuity and the likelihood of recommendations being taken to (or actioned by) senior leaders.

One senior official from Education explained that that the ability of the Education sector to dedicate more ‘space’ to nutrition in the curriculum would require a mandate from senior policy leaders, and that this would be in direct competition with other priorities, such as sexual health.

*“(Samoan) people are dying everywhere, getting sick [from NCDs]. The only way to enforce it is to have it as a policy decision from the high level. And it’s the only way to turn things around and say the focus on health must be made compulsory.... in terms of NCDs... It’s important, but it’s packed together with PE [physical education], you know?” (Senior Education Official, Interview 38)*

According to policy officers in both Health and Education, the personal motivation of individual principals to administer and enforce the Standards is a critical aspect of success.

*“It really depends on the school principal ‘cause there are principals that are really supportive, and also we’ve come across principals that just don't want to do anything with the Standards.” (Health Official, Interview 17)*

#### School-level actors are exercising ‘agency’

We found that canteen vendors were exercising a high-degree of agency in running their canteens, and that the accountability mechanisms were not adequately influencing school committees to prioritise nutrition.

*“I think they [canteen vendors] just pick the food that sells fast … they only look at income [of the canteen]… they don't see the benefits of children eating [healthy] foods” (Health Official, Interview 15)*

In at least five of the schools we visited, we noted a large disconnect between the principal’s statements of commitment to the policy, and the foods being sold. Several principals indicated that they personally had little influence over canteen sales. In two instances, the principals reported that their canteen vendor was part of the family of a leading school committee member, an arrangement that reduced the power of the principal to enforce change. One principal appeared visibly disappointed when she took us to the canteen and saw what was being sold.

*“We always talk to them [canteen vendors] about the food they bring and they sell to the pupils in school, but they don’t admire what we say” (Principal, Interview 28)*

Although every vendor we interviewed was aware of the Standards, we noted that those retailing non-compliant foods discussed so without hesitation. For instance, one vendor told us he knew of the guidelines, but had on display only cakes, cookies and sugar-sweetened beverages. Another vendor knew of the guidelines but sold only icey poles and doughnuts. None of the vendors raised with us any concerns regarding implications for non-compliance.

#### Limited capacity and resources of actors responsible for implementation

Interview participants indicated that the majority of school committees rely on revenue generated by the canteen to maintain school grounds and infrastructure, an arrangement that creates a conflict for principals attempting to enforce Standards.

Vendors in both urban and rural schools discussed that the relative cost of healthy food would make it difficult to run a successful business whilst being compliant with the Standards. One canteen manager indicated that she wasn’t committed to the Standards because the foods advocated in them were cost-prohibitive and non-competitive.

*“Cause we don't care. We’re business people, right?.…It's very expensive for the food that is required” (School Food Vendor, Interview 29)*

Vendors we met appeared to have a very limited understanding of nutrition principles, which policymakers from both Health and Education indicated was a systemic problem.

*“But now we've realised that sometimes the community find it hard to follow or to understand what constitutes healthy food” (Education Official, Interview 12)*

Though the Standards provide an extensive list of compliant foods, examples given by vendors of compliant foods indicated they were misinterpreting the guidelines. For instance, one school-based food vendor discussed how she only sold cooked noodles instead of dry noodles because they were a healthier alternative, and told us she was ‘dry frying’ commercial chicken nuggets and French fries to improve their healthfulness. Another canteen manager explained that she sold chicken franks (highly processed sausages) because they were a healthier alternative to hot dogs. One canteen manager reported converting her canteen into a healthy canteen, but was selling fries, doughnuts, cream buns, packaged biscuits, chocolate, packaged savoury snacks, dried noodles and a range of sugar-sweetened beverages.

#### The broader food environment negatively influences policy implementation

The unhealthiness of the broader food environment was consistently raised by interviewees as a problem in Samoa. The poor nutritional quality of food being sold by informal food vendors surrounding the school settings was noted by all nine of the principals we interviewed, and by half of the canteen vendors. According to interviewees, children were commonly leaving school grounds during school hours, and returning with cheap, processed foods that were likely unhealthy and would not be recommended by the Standards. The purchase of foods outside of the school environment was harming canteen viability, as well as canteen vendor commitment to implementing the Standards.

*“There’s a lot of [external] vendors now…that’s why they [school canteen operators] want to make unhealthy food because the [external] vendor is selling this food and the kids go and buy from them instead of buying the food that they are carrying in the canteen” (Policy Official, Interview 16)*

For children and their families, the affordability of healthier foods and beverages, compared with less-healthy alternatives, was consistently reported as a key barrier to the implementation of the Standards by school principals, vendors and policymakers.

*“I think despite all that we’ve been trying to do, it comes down to costs. Because a can of Coke costs sometimes go down as far as 1.50 tala [USD0.65]. And a meal, you don’t get a meal for one tala.” (Politician from the Samoa Parliamentary Advocacy Group for Healthy Lifestyles, Interview 20)*

Children’s preference for unhealthy foods, and their unwillingness to purchase healthy foods at a higher cost was perceived by both policymakers and policy implementers to be a key challenge.

#### The standards had not been formally evaluated

The Standards are monitored for compliance approximately every 6 to 12 months. Monitoring reports are routinely discussed at HPS Committee meetings, though the level of representation at these meetings reportedly limits the degree to which these are used to generate high-level policy discussion across agencies. At the time of this research, the Standards had not undergone formal evaluation. Policy officers we interviewed were unclear if compliance reports were used by policy leaders (e.g. heads of agencies and parliamentarians) to assess whether the Standards were meeting their objectives.

#### Participants’ recommendations for addressing challenges

Interviewees identified a number of opportunities to strengthen policy action. Two schools suggested that the affordability of healthy foods being recommended in the Standards needed to be revisited, and that healthier options would need to become more affordable for vendors to include them. One principal suggested others should work to engage the interest and support of community leaders and the school committee to pressure vendors operating in and around school grounds, and ban unhealthy food vendors from operating nearby. Others felt the issue of population nutrition and nutrition literacy should be addressed in the context of Samoa’s concerns around the rise of diet-related NCDs. Specifically, a number of interviewees felt that it was important to incorporate nutrition into the curriculum as a mandatory subject throughout a child’s entire primary and secondary education. It was expressed a number of times that the Standards should become fully enforceable, and that children should be banned from leaving school grounds during school hours.

## Discussion

We found health policymakers and leaders in Samoa to be strongly committed to creating nutrition-promoting environments within schools, expressed through dedication of substantial staff resources towards development, implementation and monitoring of the Standards, and the involvement of parliamentarians in school health visits. Despite this commitment, Samoa had struggled to lift compliance with the Standards and create environments that promote healthy eating within all schools. Actors both in and around the school setting were able to exercise a high degree of agency, effectively diminishing the efforts of policymakers and implementers towards successful implementation of the Standards. In particular, the food vendors and school committees reportedly had limited incentives to comply with the policy, and had a reduced capacity to do so. Actors responsible for implementation of the Standards in and around schools were limited by their nutrition literacy and financial resources, and their perception that children would not purchase healthy foods when less-healthy, more affordable alternatives were available. In Samoa, we also found that most school-based actors showed a genuine interest in the health and wellbeing of children, but were complacent about the role of food and nutrition in health, and their responsibility to promote and protect health.

Success factors for school-based food policy implementation previously identified in both higher- and lower-income countries include the need to address financial implications [14, 18, 20, 67], offer food vendors continued education, training and technical support [12, 18, 19, 22], provide educational and promotional resources to schools for use by teachers, parents and students [18, 22] and undertake regular monitoring and evaluation [18, 67, 68]. This study supports those findings, but also highlights that the creation of nutrition promoting environments in schools is likely to require a very strong mandate, powerful incentives for adoption at the local level, and meaningful sanctions for non-compliance (of a similar nature to those applied for health risks related to food safety and tobacco). Moreover, the Samoan government’s substantial implementation and monitoring efforts were not enough to generate widespread improvements to school food environments. This suggests that non-regulated (‘voluntary’) school food standards are unlikely to lead to systemic change [67].

This study also provided evidence that the unhealthiness of the broader food environment was a major problem reducing the effectiveness of school food policies [16, 24, 69]. Specific issues include the relatively high cost of healthier foods, and the widespread availability of affordable ‘junk’ food provided by informal vendors in and around schools [16, 70]. A policy restricting the sale of unhealthy foods and drinks around the school vicinity in Korea had not led to significant benefits, primarily because of food vendor concern for profitability [71]. Competitive pricing is a critical barrier to promoting healthier food options in schools [18], suggesting a need to address the relative affordability of healthy foods. Examples of such efforts could involve subsidies for healthy foods, and/or taxes on unhealthy foods [72].

In considering policy implications of this study, we identify below three key opportunities to strengthen implementation and effectiveness of policies governing the availability of food in schools, as policy learning for other settings facing similar challenges.

### Policy enforcement as an essential support mechanism

One of the important findings for strengthening multisectoral food policies, is that officials responsible for implementation find it difficult to enforce policy compliance with actors operating outside of their circle of influence (which, in the case of school food policy, includes principals, vendors, parents, students and school committees). Accordingly, powerful enforcement mechanisms, with meaningful sanctions for non-compliance, are needed to reduce pressures on actors responsible for overseeing compliance.

In the context of resource constraints that are commonly experienced in SIDS, Samoa’s integration of the Standards into Minimum Service Standards for primary and secondary schools facilitated a level of efficiency by using existing accountability mechanisms. There was opportunity to build onto this by mandating business licencing for food vendors operating in and around schools, and revoking licences from operators found not to comply. This approach has been adopted in the Australian Capital Territory (Australia), where a canteen licence is conditional on adherence to the school food and drink policy [73]. Efforts to strengthen enforcement could also draw on legally binding school food policies that have been adopted in the US [20], UK [67], Korea [71] and Brazil [74].

### Addressing policy barriers for actors responsible for implementation

Evidence suggests that policy makers may need to counter some of the financial constraints faced by individuals, schools, and communities in order to lift compliance with school food policies [14, 68, 69], for instance by reducing reliance on canteen revenue for school maintenance, or offering grants or reward programs [75]. There is also scope to facilitate programs to empower and create economic opportunities for local communities by incentivising schools to source foods from local producers [9]. Simultaneously, there is a need to reduce the influence of external food vendors on children’s food intake. The Philippines, Korea and Singapore are among countries in the Western Pacific Region that have taken steps to restrict vendors from selling unhealthy foods and beverages within a designated radius of schools [16, 17, 71].

Both our study and others [68, 71] have found that food and nutrition literacy remains low for school-level actors. Accordingly, programs to build the requisite knowledge and skills for vendors, principals, school committees and communities to implement school food policies are likely to be valuable [18, 19, 76]. Vendors may need ongoing support and training, specifically on providing low-cost, compliant food options [18]. In addition, there is evidence that school food policies that are underpinned by a clear system of food classification, including specific examples of compliant and non-compliant products can substantially reduce misinterpretation [18, 77]. Moreover, the degree to which nutrition literacy hinders the widespread adoption of school food policies, and the scale of the NCD crisis in Samoa and other SIDS, suggests that governments should consider how to address nutrition literacy more systemically [78], for instance through scaling up the volume of mandatory nutrition curriculum [79] or by offering accredited training options for food vendors and committee members [67, 80].

### Capitalising on the goodwill of political and community leaders

Consistent with previous research, this study showed that strong school food policymaking requires committed advocates at all levels, especially within the school community [21, 23, 68].

Our study and others [71, 81] have shown that actors responsible for implementation generally have a strong interest in the wellbeing of children. Developing a common purpose and building a sense of responsibility among school and community-level stakeholders may contribute to improvements in the implementation of school food policies [18], in addition to other incentives.

Formal evaluation of policy implementation is likely be helpful in engaging political and community leaders to adopt the institutional and operational commitments required to fully implement and sustain school food policies. Evaluation can extend beyond compliance monitoring by providing insights into operational aspects of a policy, the level of effectiveness in achieving policy objectives, and the value of their investments [20, 67]. Reports on policy effectiveness have reportedly played a substantial role in influencing policy and legislative change for school food in Brazil [74], the US [20] and the UK [67].

### Strengths and limitations

A key strength of this research was that we were able to interact with nearly all relevant policy officials, as well as principals and vendors from schools ranging in their compliance with the Standards. The breadth of perspectives we were able to glean was assisted by the small size of Samoa, and the relatively simple governance structures. However, due to the small number of schools involved, the insights gained may not apply universally in Samoa, and may not fully cover the range of experiences in schools across Samoa. Future research into school food policies in Samoa should seek broader input, including from students, parents, teachers and (where relevant) school committees.

To mitigate potential bias introduced through the involvement of government officials as research partners, we interviewed every policymaker deemed to have a substantial influence on the policy process. We noted that both principals and school food vendors seemed un-influenced by government officer presence and were open about challenges faced. Nevertheless, we recognize that the involvement of government officials as part of the research may have influenced the perspectives in ways we did not recognize.

As with any case study, a potential limitation to the transferability of findings is the influence of local contextual factors on policy processes. To maximize transferability beyond the Samoan context, we based our analyses on a political sciences framework that has been applied in multiple contexts [15, 63, 64]. In addition, we explicitly attempted to identify and describe local contextual factors, and focused on opportunities for drawing lessons that are likely to be applicable to other contexts.

## Conclusions

This study illustrated that the implementation of effective healthy food policies in school settings is challenging, especially when the broader food environment is not conducive to the purchase and consumption of healthier foods and beverages. Strong commitment from policy leaders is needed to create nutrition-promoting environments within schools. Even in contexts where there is political will and high-level support for healthy food policies in schools, policy dissemination, training and monitoring is unlikely to lead to successful implementation without powerful incentives for adoption at the local level and effective enforcement. This study highlighted the need for policy leaders to proactively address potential implementation issues, such as resources, capacity and accountability structures, during policy development.

This study on school food policy in Samoa has relevance for other SIDS seeking to prevent childhood obesity. In the context of limited resources, countries must prioritize nutrition-promotion within institutions with the greatest influence on children’s health and lifestyle development. There is potential to capitalize on the significant political will for preventing diet-related NCDs in many SIDS by more clearly demonstrating the institutional and operational requirements needed for effective and sustained implementation of school food policies.

## Supplementary Information

**Additional file 1:.** Interview Schedule (2017): Identifying opportunities to strengthen school food environments in the Pacific: a case study in Samoa. Interview schedule for semi-structured interviews provided in English and with Samoan translations.

## Data Availability

A restricted access to the data for this study was requested as a condition of consent for participation by Samoan research partner institutions granting ethics approval.

## Abbreviations

COREQ: Consolidated Criteria for Reporting Qualitative Research
HPAT: Health Policy Analysis Triangle
HPS: Health Promoting Schools
MESC: Ministry of Education, Sports and Culture
MOH: Ministry of Health
NCDs: Noncommunicable diseases
SIDS: Small island developing states
WHO: World Health Organization
