# Supplementary material for: Identifying opportunities to strengthen school food environments in the Pacific: a case study in Samoa
Source: BMC Public Health. 2021 Jan 29;21:246. doi: 10.1186/s12889-021-10203-2 (PMC7844953; doi:10.1186/s12889-021-10203-2)
Supplement: Supplementary file 1 — Additional file 1:. Interview Schedule (2017): Identifying opportunities to strengthen school food environments in the Pacific: a case study in Samoa. Interview schedule for semi-structured interviews provided in English and with Samoan translations. [file 12889_2021_10203_MOESM1_ESM.docx]

# Interview Schedule (2017)

### Identifying opportunities to strengthen school food environments in the Pacific: a case study in Samoa

### Erica Reeve, Christina Soti-Ulberg

#### Interview questions for policymakers (2017)

| *Thank you for agreeing to take part in this research project that forms a critical part of my PhD in public health. I know your time is extremely precious, and I really appreciate you allocating the time to contribute to this important project.*  *I am undertaking some research looking at tools to help governments to strengthen the provision, sale and marketing of healthier food options to children. The provision, sale, and marketing of food in the school environment is influenced by a great many factors, including food availability, affordability, convenience and food preferences. Food consumed at school accounts for a substantial proportion of food intake for many children, and schools are therefore an ideal setting to promote the consumption of nutritious foods.*  *You have been asked to participate as an important stakeholder in the provision of food in and around a school, other stakeholders being interviewed are policy makers, government officials, school employees, food industry and school food sellers.*  *I expect that the interview will take about 20-30 minutes. If you agree, I will handwrite notes and record the interview to make sure I accurately record your views, from the interview to make sure I accurately record your views, but please be 100% assured that all the information you provide in the interview will not be linked to your name and it will not be identifiable as having been provided by you.*  *Plain Language Statement + sign the consent form (interview + digital recording).* | |
| --- | --- |
| Policy Makers OR implementers  Ministries of Education, Health and others as relevant | - Please summarize your role in relation to school food provision - Please discuss how the Samoa School Nutrition Standards (SNS) were developed and evolved - How the SNS implemented?   - Is there an implementation or action plan (or like document)?   - Who leads implementation of the SNS and who are the key implementers? - How does implementation and monitoring of SNS differ from the other aspects of Health Promoting Schools program (e.g. environ health, food safety) - How do you promote the Samoa School Food Guidelines to schools? - Are stakeholders clear on what is allowed and not-allowed in SNS? - What are the requirements of food vendors in and around schools? (prompt: business licensing, food safety standards, adherence to SNS). - What are the complementary activities being undertaken to promote good nutrition in schools?   **Resources**  What resources have been dedicated to the sustained roll-out? (prompts: budgetary, HR, resources, tools, communication and meetings)  **Monitoring, compliance, incentives**   - How is monitoring carried out (people, tools, frequency) - Who is responsible for maintaining compliance to the SNS? (and broader HPS)   - How are the school food guidelines monitored?   - What is the implication of non-compliance? - How is compliance to the standards? - What is the primary reason for non-compliance by schools? - What ‘problem’ foods do you most often see in schools now? - Issues with current monitoring mechanism? - Compulsory versus voluntary standards under HPS (contrast Tobacco, food safety and nutrition) - What are the current incentives to adhere to SNS (versus other aspects of HPS). - Please tell us about the new principle’s performance framework, and how adherence to the guidelines is being/will be improved by that? - How much a priority are SNS versus other commitments of education sector? - Future plans for scaling up adherence to SNS (? How to make mandatory)   **Governance, coordination, oversight**   - Who would you say is the real driver/leader for improving school food? (Policy champions for SNS?) - What is your role in the HPS committee? - How does HPS committee operate and who leads/is represented? - How much influence does the HPS committee have over school food? - How difficult/easy is it to sustain interest and commitment to HPS committee? - Activity of SPAGHL and senior leadership from MESC, MOH in driving SNS. - Roles and responsibilities of school committee, community leaders and principals in maintaining SNS. |
| *Thank you again for your valuable insight into this area and for offering your time to help with this research project. Explain how I will communicate results, and Withdrawal of Consent.* | |

#### Interview questions for policymakers, parliamentarians, schools (translated by MOH Samoa)

| Introduction  Faatomuaga | *Thank you for agreeing to take part in this research project that forms a critical part of my PhD in public health. I know your time is extremely precious.*  *Faafetai mo le maliega e te auai ai i lenei suesuega . O lenei suesuega o lea faapea ona fausia ai se vaega taua i lau aoaoga maualuga i tomai faalesoifua maloloina ( PHD in Public Health). Ou te faafetaia foi le faaavanoaina o lou taimi o mo lenei suesuega.*  *I am undertaking some research looking at tools to help governments to strengthen the provision, sale and marketing of healthier food options to children. The provision, sale, and marketing of food in the school environment is influenced by a great many factors, including food availability, affordability, convenience and food preferences.*  *O lea faapea ona ou suesueina ni auala poo metotia e fesoasoani tele ai i le malo i le unaia o le faatau atu ma le faalauiloaina o taumafa e soifua maloloing mo a tatou fanau. O ituaiga meaai e faatau atu , faalauiloaina ma iai i totonu o aoga e mafua ona o le tele o ni vaega eseese e pei o le itu i le maua gofie, maua i taimi uma, taugofie, faigofie ma le manaoga/filifiliga lava o le tagata.*  *You have been asked to participate as an important stakeholder in the provision of food in and around a school, other stakeholders being interviewed are policy makers, government officials, school employees, food industry and school food sellers.*  *Ua filifiliaina oe e auai i lenei suesuega ona o lou tomai ma agavaa e uiga i meaai i totonu o aoga, o nisi tagata ua filifiliaina i lenei suesuega o tagata faitulafono, tagata faigaluega a le malo, e o faafaigalueina i totonu o aoga, fale gaosi meaai ma tagata faipinisi laiti e pei o e e faatau atu meaai i tamaiti aoga.*  *I expect that the interview will take about 20-30 minutes. If you agree, I will handwrite notes and record the interview to make sure I accurately record your views. All the information you provide in the interview will not be linked to your name and it will not be identifiable as you*  *Ou te talitonu e tusa ma le 20-30 minute le umi o lenei faatalatalanoaga. Afai e te malie iai, o lea ou faamauina i lalo ma pueina i le masini pu’e leo lenei faatalatalanoaga. Ó faamatalaga uma o lea tuuina mai i lenei faatalatalanoaga e le faamauina ai lou suafa pe faailogaina ai lou tagata.* |
| --- | --- |
| Policy Makers  OR  Department of Education bureaucrats  O e o loo fausiaina faiga faavae  Poo le Matagaluega o Aoga. | - Please discuss the Samoa School Food Guidelines   Faamolemole pe mafai ona e faamatalaina mai Taiala o Taumafa Tatau i totonu o Aoga   - - How were the Samoa School Food Guidelines developed?   - Sa faapefea ona fausiaina nei Taiala o Taumafa Tatau i totonu o Aoga   - Who was involved?   - O ai ni tagata na fausiana nei Taiala?   - How they are implemented?   - E faapefea ona faaaogaina nei taiala i totonu o aoga?   - Is there an oversight committee, and how often do they meet?   - O iai se komiti faapitoa e vaavaaia uma tulaga o lenei taiala ma pe faafia foi ona latou fono?   - Is there an implementation plan (or like document)?   - O iai se fuafuaga fa’ataatia ile fa’atinoina o galuega fai o lenei Ta’iala? ( poo se tusi e faamatalaina ai ni taiala o taumafa mo aoga)?   - What resources have been dedicated to the sustained roll-out?   - O a ni oloa/ ????? e faaaogaina mo le faaauauina o nei taiala i totonu o aoga? - How do you promote the Samoa School Food Guidelines to schools? - E faafefea ona e faalauiloaina Taiala o Taumafa Tatau i Totonu o Aoga? - Who in Government is responsible for implementing and monitoring? - O ai tagata faigaluega a le malo/ Matagaluega o le Malo ua filifilia e faaogaina pe tulimataia nei Taiala?   - How are the school food guidelines monitored?   - E faapefea ona tulimataia le taiala i totonu o aoga?   - What happens to schools not complying?   - O a ni tulaga e faatinoina i aoga e le mulimulitai i nei taiala mo aoga? - Please tell us about the principle’s performance framework, and how adherence to the guidelines is being improved by that? - Faamolemole pe mafai ona e faamatalaina mai ni Aiaiga poo faiga faavae o galuega e faatinoina e Pule Aoga ma lona fesootaiga i Taiala o Taumafa Tatau i Totonu o Aoga? - What is the primary reason for non-compliance by schools? - O le a le mafuaaga maumaututu o lo’o iai e le o mafai ai ona ausia e aoga nei Taiala? |
| SPAGHL member-  Parliamentary advocacy group  Komiti Faapalemene e unaia faiga faavae mo le Soifua Maloloina i Samoa. | - Can you tell me about SPAGHL’s role in implementing healthy eating in schools? - E mafai ona e faamatalaina mai le galuega a le “ Komiti Faapalemene e unaia faiga faavae mo le Soifua Maloloina i Samoa” o lo’o faia i le unaia o le taumafaina o taumafa tatau i totonu o aoga? - How healthy have you found school food to be? - O lea sou silasila i le soifua maloloina o meaai i totonu o aoga ? - What has been SPAGHL’s ongoing role in monitoring? - O a galuega a lenei Komiti o loo faia i le tulimataia o meaai talafeagai i totonu o aoga? - What other food policies is SPAGHL championing? - O a nisi faiga faavae i taumafa o loo faapea ona unaia malosi e lenei Komiti Faapalemene?   - Is there interest for further collaboration?   - O iai se naunautaiga e faaauauina e le lumanai galuega a lenei Komiti i galuega faasoifua maloloina? |
| School leaders (i.e. Principal, senior teaching staff)  Taitai o Aoga ( ft. Pule Aoga, Senia o le Aoga, Faiaoga ma isi)++* | - How do children procure FNAB at your school *(i.e. purchase inside school, bring own food etc)* - E faapefea ona faatau e tamaiti meaai i le tou aoga ( ft. Faatau meaai i totonu o aoga, aumai meaai mai le fale pe faatau i fafo o le aoga) - Who determines what FNAB are provided/sold/obtained by children? - O ai e faia fa’ai’uga fai i ituaiga meaai ma vaiinu e faatau pe taumafaina i totonu o le aoga? - What do you know about the Samoa School Food Guidelines? - O lea sou malamalamaaga i le Taiala o Taumafa Tatau mo Aoga? - What is the biggest challenge with following the Samoa School Food Guidelines? - O a ni luitau e pito i sili ona faigata ai ona mulimuli tai i lenei Taiala?   **Where children bring own food- Mo tamaiti o loo aumai meaai i le aoga.**   - What type of FNAB are commonly brought by children? - O a ni ituaiga meaai e masani ona aumai e tamaiti i le aoga? - Are these purchased nearby the school (where)? - E faatau nei meaai i se faleoloa e lata ma i aoga ( o fea)? - Are there any restrictions on what FNAB children can bring into the school? - E iai ni meaaiga e le faatagaina ona aumai e tamaiti i le aoga? - Do you provide any guidance to children or parents on school-suitable foods? - E te aveina ni faamatalaga i tamaiti aoga poo matua i meaai e talafeagai mo aoga?   **Where school provides or sells food- Mo aoga e faatau ai meaai**   - How are you using the Samoa School Food Guidelines in your school? - O faapefea ona e faaogaina le Taiala o Taumafa Tatau mo Aoga i lou aoga? - What types of (‘good’ and ‘bad’) foods are commonly sold/provided? - O a ni meaai ( lelei pe leaga) e masani on faatau ai? - What are some of the problems you would have in implementing healthy FNAB policies? - O a ni faafitauli e ono iai i le faatinoina o ni faiga faavae mo taumafa paleni i totonu o aoga? - *(prompts only if needed, including additional preparation time required, affordability, availability, skill level, human resources)* - *(nisi fesili pe a manaomia, taimi e gaseseina ai meaai, taugofie o taumafa, maua gofie o taumafa, le maualuga o agavaa, oloaina i???? tagata faigaluega e faatinoina nei vaega)* - Do you consider that it to be important that healthier foods are provided over unhealthy, in the context of your priorities at the school? - E te manatu e taua le soifua maloloina o taumafa i aoga, pe a e faatulagaina nisi faamoemoe e ausia e lou aoga?   **Where children purchase from a third-party provider (e.g. hawker, private vendor)-**  **Mo tamaiti e faatauina taumafa mai nisi faleaiga laiti. (ft. Faleaiga faasavali, pisini laitI tumaoti)**   - Do you have in your control to regulate the types of foods sold *inside* the school grounds? - E iai sou malosiaga e taofiina ai meaai e faatau i totonu o le lotoa o le aoga?   - How do you do this?   - E faapefea ona e faia lenei tulaga? - Do you have in your control to regulate the types of foods sold *around* the school grounds? - E iai sou malosiaga e taofiina ai meaai e faatauina lata mai le aoga? - Is the school able to work with private providers to bring them into line with healthy food guidelines? - O mafai e le aoga ona galulue faatasi ma pisinisi tumaoti e o gatasi ai meaai o loo faatauina ma Taiala o Taumafa Tatau mo Aoga?   **On food marketing- Faalauiloa i Aoga**   - Do any FNAB-related companies provide the school with any support (ie funding, sponsorship, sports events, fundraising activities, freebies, gifts, equipment) - O foaaina mai e kamupani e gaosiina meaai ma vaiinu le talafeagai ni fesoasoani mo le aoga?   (ft. Faatupeina o taaloga, suega tupe mo le aoga, meaai fua, masini mo le aoga, meaalofa mo le aoga)   - Are there any posters, billboards, fliers marketing FNAB inside the school? - O iai ni siata , laupapa o faamatalaga , pepa o faamatalaga e faalauiloa ai oloa taumafa o nei kamupani i totonu o le aoga? |
| Food Vendors | - Does the school provide you with direction/guidelines on what foods you provide/sell to children? - E foaiina mai e le aoga faatonuga/taiala i meaai e foai / faatau atu i tamaiti aoga? - What kinds of *foods* are the most commonly sold/provided (document 7) - O a ituaiga meataumafa e masani ona faatau atu pe foai i le aoga ( pepa faamatalaga e 7) - What kinds of *drink* are the most commonly sold/provided (document 4) - O a ituaiga vaiinu e masani ona faatau atu/ pe foai i le aoga ( pepa o faamatalaga e 4) - What are some of the ‘healthier’ local foods that you could potentially sell? - O a ni meaai mai o tatou laufanua e soifua maloloina le gaseseina e te iloa e mafai ona e faatauina? - What would be the key problems that would make it difficult to prepare and sell these foods? - O a ni faafitauli e te iloa e faafaigata ai ona e gaseseina nei mea taumafa pe faatauina atu? - *(prompts include accessing regularly, affordability, children may not like/purchase the foods, it would require more time/skill to prepare, transport)?* - *(nisi fesili o le maua gofie, taugofie, e le fiafia ai tamaiti i meaai/faatau mai meaai, e tele atu le taimi/agavaa moomia e gaseseina ai ma kiliva a ii le aoga)* |
| Thank you | *Thank you again for your valuable insight into this area and for offering your time to help with this research project.*  *E toe momoli atu le faafetai mo faamatalaga ua faapea ona tuuina mai e uiga i lenei mataupu. Faafetai foi mo le faaavanoaina o lou taimi e fesoasoani mai a i lenei poloketi/suesuega).*  *Explain how I will communicate results, and Withdrawal of Consent.*  *Faamatala auala o le faaooina atu ai faamaumauga o lenei suesuega pea a maea ona faatautaia ma nisi tulaga e Pea le toe fia auai se tagata i lenei suesuega.* |
